# Supplementary material for: Effects of digital chatbot on gender attitudes and exposure to intimate partner violence among young women in South Africa
Source: PLOS Digit Health. 2023 Oct 16;2(10):e0000358. doi: 10.1371/journal.pdig.0000358 (PMC10578594; doi:10.1371/journal.pdig.0000358)
Supplement: S1 Fig — (DOCX) [file pdig.0000358.s001.docx]

S1 Fig. Consent flow

During the onboarding process, users received information about the intervention and the research study. After users clicked on a Facebook advertisement on their device, they were directed to WhatsApp. On WhatsApp, users were exposed to a brief description of the trial with all the key information required for consent. The user then received a PDF explaining the nature of the trial in plain terms. The user was asked if they wanted to participate in the trial; if they agreed, they were allocated to a trial arm at random using a built-in algorithm.


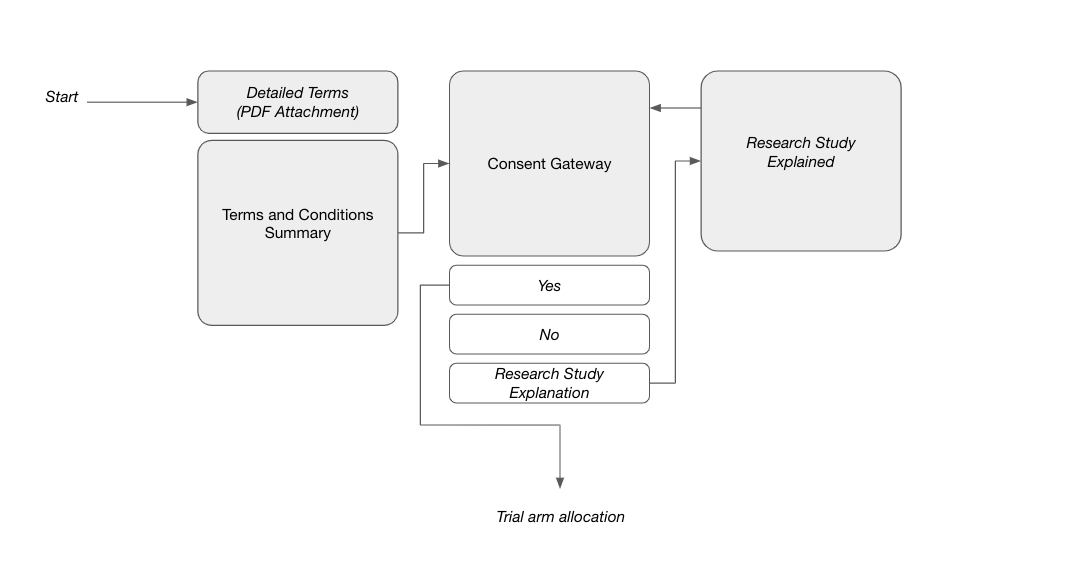


The user was automatically remunerated with preloaded airtime after the completion of the primary outcome quiz. Users who could not receive automated airtime remunerations were sent money vouchers, redeemable as cash or airtime at any bank ATM, manually via SMS.
