# Supplementary figures and images for: Effects of digital chatbot on gender attitudes and exposure to intimate partner violence among young women in South Africa
Source: PLOS Digit Health. 2023 Oct 16;2(10):e0000358. doi: 10.1371/journal.pdig.0000358 (PMC10578594; doi:10.1371/journal.pdig.0000358)

S2 Figure. Mental health by trial arm, *n=*5,022


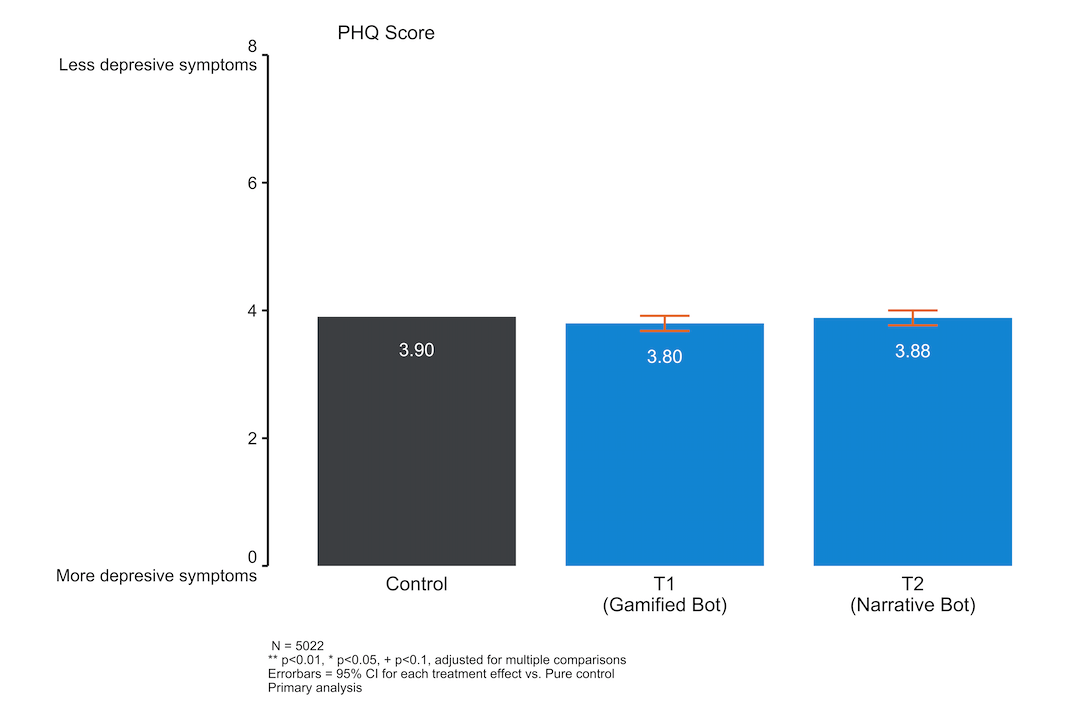

Supplement: S2 Fig — (DOCX) [file pdig.0000358.s002.docx]
