# Supplementary material for: Effects of digital chatbot on gender attitudes and exposure to intimate partner violence among young women in South Africa
Source: PLOS Digit Health. 2023 Oct 16;2(10):e0000358. doi: 10.1371/journal.pdig.0000358 (PMC10578594; doi:10.1371/journal.pdig.0000358)
Supplement: S1 Table — (DOCX) [file pdig.0000358.s003.docx]

S1 Table. Intervention modules

| **Module** | **Challenge** | **Module aim** |
| --- | --- | --- |
| **Module 1** | **Signs of IPV are often justified, or poorly identified**  Qualitative and observational studies in South Africa show that IPV is pervasive ^^[[1]](#endnote-1)^^ ^^[[2]](#endnote-2)^^ and normalised.^^[[3]](#endnote-3)^^ In that context, unhealthy relationship behaviours are often justified or overlooked and there is limited nuance in young women’s understanding of controlling, coercive or abusive behaviours, particularly in the private realm.^^[[4]](#endnote-4)^^ For example, emotional or financial abuse (including jealousy and controlling behaviours) are often confused for normal relationship conflict or even for protective and loving attitudes. | **Identify unhealthy relationship behaviours**  In T1, the user is presented with a series of scenarios and is then asked to determine if the illustrated situation is healthy or unhealthy. She then receives immediate and iterative feedback.^^[[5]](#endnote-5)^^ In gamified approaches, users are given a safe space to try out new skills and receive structured feedback, which can improve learning outcomes.^^[[6]](#endnote-6)^^ Applied to sexual and repruductive health, studies have shown that gamified designs can improve knowledge and identification outcomes,^^[[7]](#endnote-7)^^ as well as self-efficacy.^^[[8]](#endnote-8)^^  In T2, the user is introduced to the concept of healthy or unhealthy relationship behaviours indirectly through the narrative. Users are also prompted to engage critically by identifying if behaviours within the narrative are healthy or unhealthy. |
| **Module 2** | **Limited social support**  Social support is associated with positive mental health outcomes and reduced exposure to violence for women experiencing IPV.^^[[9]](#endnote-9)^^ ^^[[10]](#endnote-10)^^ Qualitative research emphasises the importance of young women’s reliance on friends or trusted adults to reduce their exposure to violence.^^[[11]](#endnote-11)^^ When supported by peers or family, girls feel more comfortable exerting autonomy or agency in relationships.^^[[12]](#endnote-12)^^ | **Identify a trusted person to speak to about relationship**  In T1, ChattyCuz describes the characteristics of a trusted person for the user, using emojis. Users follow prompts to identify supportive traits or unhelpful responses by potential trusted friends, and receive feedback on their choices. By working through the questions, the user earns the One You Can Always Trust badge.  In T2, users hear from Sandiswa as she looks for a trusted friend, finding some who provide unhelpful advice and persevering until she finds someone who listens carefully to her experiences and makes her feel safe. The chatbot then reaffirms the characteristics of a trusted person, using the same characteristics outlined in T1. |
| **Module 3** | **Low autonomy and self-efficacy**  Correlational studies in rural South Africa link IPV to a lack of autonomy, defined as freedom and agency in life situations.^^[[13]](#endnote-13)^^ ^^[[14]](#endnote-14)^^ Self-efficacy, defined as the belief that one can voice their opinions or stand up to mistreatment,^^[[15]](#endnote-15)^^ Affirmation exercises to reflect positively self-worth in intimate relationships, learning confidence, and seeking help or helping others are all skills-based ways to improve self-efficacy.^^[[16]](#endnote-16)^^[[17]](#endnote-17)^^ | **Skills to improve self-efficacy**  In T1, ChattyCuz asks the user to reflect and answer a series of questions about their relational values and their sources of power, in order to earn the Helping Hand badge.  In T2, Sandiswa explains that Jabu’s behaviour has upset her and that she wants to be kind to herself. Drawing a connection between Sandiswa’s desire to be kinder to herself and the experience of the user, ChattyCuz then asks the user to reflect on questions about their relational values and sources of power. |
| Module 4 | **Limited understanding of healthy communication skills in my intimate relationships**  IPV reduction is more likely to occur in contexts where both partners improve conflict resolution skills and where societal norms around the legitimacy of violence as a tool for communication are actively challenged.  Developing new skills around healthy and respectful communication and decision-making is an important prerequisite for IPV-related behaviour change,^^[[18]](#endnote-18)^^ and studies show that shifting relationship communication from male-led to joint-decision making strengthens partner relationships.^^[[19]](#endnote-19)^^ ^^[[20]](#endnote-20)^^ | **Practice healthy communication skills for relationships**  This module aims to teach users about healthy communication and conflict resolution skills in relationships and to give users the chance to practice them. Content is designed to support users to understand what healthy communication looks like, to identify unhealthy communication traits in their partners, and to respond in the most effective and safe manner possible.  In T1, users answer a series of questions focusing on communication skills that should be practised by both partners, including active listening skills, maintaining respect for boundaries, and expressing feelings safely to earn a Super Communicator badge.  In T2, users hear about two conversations between Sandiswa and Jabu, one in which they had a fight in front of other people and a more productive conversation. Users get an opportunity to suggest what Sandiswa should do next. ChattyCuz then explains that open, honest communication is an important part of a healthy relationship. |
| Module 5 | **An action-intention gap preventing safety seeking**  People sometimes fail to follow through on their intentions because they procrastinate (e.g. putting off calling a help centre), miss the opportunity to act (e.g. failing to call the helpline when they have a moment alone), or have second thoughts at a critical moment (e.g. deciding against leaving an unhealthy relationship at the last moment). This phenomenon is referenced in behavioural science literature as an intention-action gap.^^[[21]](#endnote-21)^^  Safety planning is used to help advocates and women talk through strategies that would increase safety and create plans to action them. A systematic review of existing studies suggests that women in IPV situations use a variety of safety strategies to protect themselves.^^[[22]](#endnote-22)^^ Evidence presented in this review shows modest support for the use of safety planning over resistance strategies, which appear to show a greater risk of re-victimisation for those women who use them.^^[[23]](#endnote-23)^^ The results of one study in South Africa suggest that using planning protocols may reduce IPV for women who engage in activities that might place them at more risk of violence from their partners, such as visiting an HIV-testing unit.^^[[24]](#endnote-24)^^ | **Make a safe plan**  In T1, users are presented with three subtopics that they can choose to discuss with ChattyCuz: making a plan to stay in a relationship, making a plan to leave a relationship, or links to a support helpline. To earn the Safety Expert badge, the user needs to explore the first two topics.  In T2, users can suggest whether Sandiswa should stay with Jabu or leave him. According to their choice, they then hear a story in which Sandiswa makes a plan to leave and does so safely, or one in which she stays but makes a safe plan to leave in the future. Both arms of the narrative provoke a discussion about the characteristics of a safe plan. |
| Module 6 | **Limited psychological support during difficult experiences**  A study looking at links between IPV and suicidality demonstrated that suicide attempters were those who showed less adaptive (alcohol or drug abuse) coping strategies, while non-attempters engaged in more positive coping strategies (religion and community resources, social support).^^[[25]](#endnote-25)^^ Longitudinal evidence suggests that using positive, active coping mechanisms is generally predictive of positive mental health, while disengagement strategies, like avoiding problems, are predictive of poorer mental health.^^[[26]](#endnote-26)^^ ^^[[27]](#endnote-27)^^ | **Identify practical coping mechanisms for difficult experiences**  This module aims to build knowledge about positive coping mechanisms.  In T1, ChattyCuz engages with the user in a conversation about coping strategies to use when feeling stressed or anxious. In order to earn the Stay Calm Queen badge, gamified bot users need to answer a series of true or false questions about the best way to cope.  In T2, ChattyCuz presents the final instalment of Sandiswa’s story, in which the user learns how Sandiswa is coping with stress and anxiety. ChattyCuz reminds the user about various coping strategies to deal with stressful situations and invites the user to type out strategies that she likes to use. |
| Content integrated across modules | | |
| **All modules** | **Limited awareness of power imbalance and control in relationships**  Observational studies from South Africa and around the world have found direct associations between autonomy, beliefs about gender equality and IPV exposure among young women, suggesting that positive power equity beliefs may be protective against IPV.^^[[28]](#endnote-28)^^ ^^[[29]](#endnote-29)^^ ^^[[30]](#endnote-30)^^ Additionally, low relationship power and IPV are also associated with high HIV risks.^^[[31]](#endnote-31)^^ | **Encourage reflections about sharing power and maintaining autonomy in relationships**  Programs like SASA! have shown the promise of power-based approaches and critical reflection on harmful norms to reduce IPV.^^[[32]](#endnote-32)^^  ChattyCuz invites users to reflect critically about power imbalance and control in relationships through structured quizzes and conversations, or narrative-based content.  Research shows the need to resonate with the lived realities of women they target.^^[[33]](#endnote-33)^^ For that reason, T1 and T2 incorporate relevant examples throughout, reflecting findings from qualitative work on how women experience IPV in South Africa. |

1. George, E. (2001). Scared at School: Sexual Violence Against Girls in South African Schools. Publications Department, Human Rights Watch, 350 Fifth Avenue, 34th Floor, New York, NY 10018-3299. [↑](#endnote-ref-1)
2. Sibanda-Moyo, N., Khonje, E., & Brobbey, M. K. (2017). Violence against women in South Africa: A country in crisis 2017. [↑](#endnote-ref-2)
3. Thaler, K. (2012). Norms about intimate partner violence among urban South Africans: A quantitative and qualitative vignette analysis. [↑](#endnote-ref-3)
4. Makongoza, M., & Nduna, M. (2021). Awareness and rejection accounts of intimate partner violence by young women in Soweto, Johannesburg, South Africa. Journal of interpersonal violence, 36(1-2), 7-32. [↑](#endnote-ref-4)
5. Butler, M., Pyzdrowski, L., Goodykoontz, A. and Walker, V., 2008. The effects of feedback on online quizzes. International Journal for Technology in Mathematics Education, 15(4) [↑](#endnote-ref-5)
6. Hamari, J., Koivisto, J. and Sarsa, H., 2014, January. Does Gamification Work?-A Literature Review of Empirical Studies on Gamification. In HICSS (Vol. 14, No. 2014, pp. 3025-3034). [↑](#endnote-ref-6)
7. Datta, S., Burns, J., Maughan-Brown, B., Darling, M., & Eyal, K. (2015). Risking it all for love? Resetting beliefs about HIV risk among low-income South African teens. Journal of Economic Behavior & Organization, 118, 184-198. [↑](#endnote-ref-7)
8. Thomas, R., Cahill, J. and Santilli, L., 1997. Using an interactive computer game to increase skill and self-efficacy regarding safer sex negotiation: Field test results. Health Education & Behavior, 24(1), pp.71-86. [↑](#endnote-ref-8)
9. Sabri, B., Simonet, M. and Campbell, J.C., 2018. Risk and protective factors of intimate partner violence among South Asian immigrant women and perceived need for services. Cultural diversity and ethnic minority psychology, 24(3), p.442. [↑](#endnote-ref-9)
10. "Posttraumatic stress disorder among battered women: risk and ...." <https://www.ncbi.nlm.nih.gov/pubmed/8292561>. Accessed 24 Apr. 2020. [↑](#endnote-ref-10)
11. Ibid. [↑](#endnote-ref-11)
12. Willan, S., Ntini, N., Gibbs, A., & Jewkes, R. (2019). Exploring young women’s constructions of love and strategies to navigate violent relationships in South African informal settlements. Culture, health & sexuality, 21(11), 1225-1239. [↑](#endnote-ref-12)
13. Mpondo, F., Ruiter, R.A., van den Borne, B. and Reddy, P.S., 2016. Intimate partner violence and its association with self-determination needs and gender-power constructs among rural South African women. Journal of interpersonal violence, p.0886260516664316. [↑](#endnote-ref-13)
14. Zembe, Y.Z., Townsend, L., Thorson, A., Silberschmidt, M. and Ekstrom, A.M., 2015. Intimate partner violence, relationship power inequity and the role of sexual and social risk factors in the production of violence among young women who have multiple sexual partners in a peri-urban setting in South Africa. PloS one, 10(11), p.e0139430. [↑](#endnote-ref-14)
15. Sabatelli,R., Anderson, S., Trachtenberg, J, and Liefeld, J. (2005), adapted from Bandura (2006) Self-Efficacy Beliefs of Adolescents. Pages 307–337. [↑](#endnote-ref-15)
16. Bandura, A. 1986. Chapter 9: Self Efficacy. In Social foundations of thought and action: A social cognitive theory (pp. 390-453). Upper Saddle River, NJ: Prentice Hall. [↑](#endnote-ref-16)
17. Cohen, G. L., & Sherman, D. K. (2014). The psychology of change: Self-affirmation and social psychological intervention. Annual review of psychology, 65, 333-371. [↑](#endnote-ref-17)
18. "Change Among Batterers: Examining Men's Success Stories ...." <https://journals.sagepub.com/doi/10.1177/088626000015008003>. Accessed 24 Apr. 2020. [↑](#endnote-ref-18)
19. "Change Among Batterers: Examining Men's Success Stories ...." <https://journals.sagepub.com/doi/10.1177/088626000015008003>. Accessed 24 Apr. 2020. [↑](#endnote-ref-19)
20. Russell, M., Cupp, P. K., Jewkes, R. K., Gevers, A., Mathews, C., LeFleur-Bellerose, C., & Small, J. (2014). Intimate partner violence among adolescents in Cape Town, South Africa. Prevention Science, 15(3), 283-295. [↑](#endnote-ref-20)
21. Parks–Stamm, E.J., Gollwitzer, P.M. and Oettingen, G., 2007. Action control by implementation intentions: Effective cue detection and efficient response initiation. Social Cognition, 25(2), pp.248-266. [↑](#endnote-ref-21)
22. Parker, E. M., & Gielen, A. C. (2014). Intimate partner violence and safety strategy use: frequency of use and perceived effectiveness. Women's health issues, 24(6), 584-593. [↑](#endnote-ref-22)
23. Parker, E. M., & Gielen, A. C. (2014). Intimate partner violence and safety strategy use: frequency of use and perceived effectiveness. Women's health issues, 24(6), 584-593. [↑](#endnote-ref-23)
24. Brown, L.L. and Van Zyl, M.A.R., 2018. Mitigating intimate partner violence among South African women testing HIV positive during mobile counseling and testing. AIDS Care, 30(1), pp.65-71. [↑](#endnote-ref-24)
25. Reviere, S. L., Farber, E. W., Twomey, H., Okun, A., Jackson, E., Zanville, H., & Kaslow, N. J. (2007). Intimate partner violence and suicidality in low-income African American women: A multimethod assessment of coping factors. Violence against women, 13(11), 1113-1129. [↑](#endnote-ref-25)
26. Taft, C. T., Resick, P. A., Panuzio, J., Vogt, D. S., & Mechanic, M. B. (2007). Coping among victims of relationship abuse: A longitudinal examination. Violence and victims, 22(4), 408-418. [↑](#endnote-ref-26)
27. Itimi, K., Dienye, P. O., & Gbeneol, P. K. (2014). Intimate partner violence and associated coping strategies among women in a primary care clinic in Port Harcourt, Nigeria. Journal of family medicine and primary care, 3(3), 193. [↑](#endnote-ref-27)
28. Stöckl, H., March, L., Pallitto, C., & Garcia-Moreno, C. (2014). Intimate partner violence among adolescents and young women: prevalence and associated factors in nine countries: a cross-sectional study. BMC public health, 14(1), 751. [↑](#endnote-ref-28)
29. Mpondo, F., Ruiter, R. A., van den Borne, B., & Reddy, P. S. (2019). Intimate partner violence and its association with self-determination needs and gender-power constructs among rural South African women. Journal of interpersonal violence, 34(14), 2975-2995. [↑](#endnote-ref-29)
30. Russell, M., Cupp, P. K., Jewkes, R. K., Gevers, A., Mathews, C., LeFleur-Bellerose, C., & Small, J. (2014). Intimate partner violence among adolescents in Cape Town, South Africa. Prevention Science, 15(3), 283-295. [↑](#endnote-ref-30)
31. Teitelman, A. M., Jemmott III, J. B., Bellamy, S. L., Icard, L. D., O'Leary, A., Heeren, G. A., ... & Ratcliffe, S. J. (2016). Partner violence, power, and gender differences in South African adolescents’ HIV/sexually transmitted infections risk behaviors. *Health psychology*, *35*(7), 751. [↑](#endnote-ref-31)
32. Abramsky, T., Devries, K.M., Michau, L., Nakuti, J., Musuya, T., Kyegombe, N. and Watts, C., 2016. The impact of SASA!, a community mobilisation intervention, on women's experiences of intimate partner violence: secondary findings from a cluster randomised trial in Kampala, Uganda. J Epidemiol Community Health, 70(8), pp.818-825. [↑](#endnote-ref-32)
33. Gibbs, A. (2016). Tackling gender inequalities and intimate partner violence in the response to HIV: moving towards effective interventions in Southern and Eastern Africa. African Journal of AIDS Research, 15(2), 141-148. [↑](#endnote-ref-33)
