# Supplementary material for: Effects of digital chatbot on gender attitudes and exposure to intimate partner violence among young women in South Africa
Source: PLOS Digit Health. 2023 Oct 16;2(10):e0000358. doi: 10.1371/journal.pdig.0000358 (PMC10578594; doi:10.1371/journal.pdig.0000358)
Supplement: S2 Table — (DOCX) [file pdig.0000358.s004.docx]

S2 Table. Gender equitable beliefs regression

|  | ***1*** | ***2*** | ***3*** | ***4*** |
| --- | --- | --- | --- | --- |
|  | **GRS score** | **GRS score** | **GRS score** | **GRS score** |
|  | **OLS** | **OLS** | **OLS** | **CEM** |
| **Attention Control (T0)** | 0.70** | 0.54** | 0.50** | 0.43* |
|  | -0.18 | -0.17 | -0.17 | -0.19 |
| **ChattyCuz-Gamified (T1)** | 1.66** | 1.47** | 1.49** | 1.50** |
|  | -0.12 | -0.11 | -0.11 | -0.12 |
| **ChattyCuz-Narrative (T2)** | 1.08** | 0.97** | 0.99** | 0.96** |
|  | -0.12 | -0.11 | -0.11 | -0.12 |
| **Baseline attitudes in regression** | N | Y | Y | N |
| **Additional controls in regression^** | N | N | Y | N |
| **Constant** | 18.55** | 14.49** | 14.25** | 18.58** |
|  | -0.08 | -0.16 | -0.28 | -0.08 |
| **Observations** | N=11,630 | N=11,519 | N=11,519 | N=11,447 |
| + p < 0.10, * p < 0.05, ** p < 0.01 D32 | |  |  |  |
| ^ additional controls are: age, mental health at baseline, partnership status | | | | |
