# Supplementary material for: Effects of digital chatbot on gender attitudes and exposure to intimate partner violence among young women in South Africa
Source: PLOS Digit Health. 2023 Oct 16;2(10):e0000358. doi: 10.1371/journal.pdig.0000358 (PMC10578594; doi:10.1371/journal.pdig.0000358)
Supplement: S3 Table — (DOCX) [file pdig.0000358.s005.docx]

S3 Table. Any IPV exposure regression

|  | ***1*** | ***2*** | ***3*** |
| --- | --- | --- | --- |
|  | **Unadjusted** | **Adjusted for baseline attitudes** | **Adjusted for baseline attitudes and controls^** |
| **ChattyCuz-Narrative**  **(T2)** | -0·02 | -0.02 | -0.02 |
|  | (0·02) | -0.02 | -0.02 |
| **ChattyCuz-Gamified (T1)** | -0.06^**^ | -0.06^**^ | -0.07^***^ |
|  | -0.02 | -0.02 | -0.02 |
| **Observations** | N=4139 | N=4094 | N=3955 |
| ^***^p < 0.001; ^**^p < 0.01; ^*^p < 0.05 | | | |
| ^ additional controls are: age, mental health at baseline, partnership status, food security, household composition, number of children | | | |
