# Supplementary material for: Effects of digital chatbot on gender attitudes and exposure to intimate partner violence among young women in South Africa
Source: PLOS Digit Health. 2023 Oct 16;2(10):e0000358. doi: 10.1371/journal.pdig.0000358 (PMC10578594; doi:10.1371/journal.pdig.0000358)
Supplement: S4 Table — (DOCX) [file pdig.0000358.s006.docx]

| S4 Table. Technological and/or psychological IPV | | | |
| --- | --- | --- | --- |
|  | **(1)**  **Unadjusted** | **(2)**  **Adjusted for baseline attitudes** | **(3)**  **Adjusted for baseline attitudes and controls^** |
|  |  |  |  |
| **ChattyCuz-Narrative (T2)** | -0·05^*^ | -0·05^*^ | -0·05^**^ |
|  | (0·02) | (0·02) | (0·02) |
| **ChattyCuz-Gamified (T1)** | -0·07^***^ | -0·07^***^ | -0·08^***^ |
|  | (0·02) | (0·02) | (0·02) |

^***^p < 0.001; ^**^p < 0.01; ^*^p < 0.05

^ additional controls are age, mental health at baseline, and partnership status

While 26% of young women in control reported past-month exposure to physical and/or sexual violence, the rates T2 is similar (27%) and while the estimate for T1 is lower (24%), this does not reach statistical significance.
