# Supplementary material for: Effects of digital chatbot on gender attitudes and exposure to intimate partner violence among young women in South Africa
Source: PLOS Digit Health. 2023 Oct 16;2(10):e0000358. doi: 10.1371/journal.pdig.0000358 (PMC10578594; doi:10.1371/journal.pdig.0000358)
Supplement: S5 Table — (DOCX) [file pdig.0000358.s007.docx]

| S5 Table. Physical and/or sexual IPV | | | |
| --- | --- | --- | --- |
|  | **(1)**  **Unadjusted** | **(2)**  **Adjusted for baseline attitudes** | **(3)**  **Adjusted for baseline attitudes and controls^** |
| **ChattyCuz-Narrative (T2)** | 0.01 | 0.01 | 0.01 |
|  | (0.02) | (0.02) | (0.02) |
| **ChattyCuz-Gamified (T1)** | -0.02 | -0.02 | -0.02 |
|  | (0.02) | (0.02) | (0.02) |
| ^ additional controls are age, mental health at baseline, and partnership status | | | |
