# Supplementary material for: Effects of digital chatbot on gender attitudes and exposure to intimate partner violence among young women in South Africa
Source: PLOS Digit Health. 2023 Oct 16;2(10):e0000358. doi: 10.1371/journal.pdig.0000358 (PMC10578594; doi:10.1371/journal.pdig.0000358)
Supplement: Attachment — Submitted filename: Response to reviewer comments 20.docx [file pdig.0000358.s008.docx]

Dear PLoS Digital Health,

It is a pleasure to resubmit a revised version of our manuscript. Please find, below, point-by-point responses to reviewer comments.

Warm regards,

Authors of PDIG-D-22-00354

**Reviewer #1: I would like to thank the publisher and the journal for involving me in the peer review of this very interesting study on the “Effects of digital chatbot on gender beliefs and exposure to intimate partner violence among young women in South Africa”.**

**The study is conducted with great scientific attention and deals with a level of prevention and promotion of crucial importance for people's health and particularly for young women who may be victims of IPV. However, in order for the study to be even more accessible and interesting, some points of reflection are suggested (Note: in the absence of the number of lines, the name of the chapter, paragraph, any SUB, and the line number within the cited chapter, paragraph or sub are indicated: for example, "Abstract", paragraph "Methods", LINE 2 does not refer to line 2 of the entire document or page, but to line 2 of the "Methods" paragraph):**

1. **"Abstract", paragraph "Methods", LINE 2, in addition to the number indicated as recruited people "(n = 19,643)" it would be interesting to know, in principle, also the number of people who actually participated in the clinical study (n = 11,630) and the relationship that exists between these two numbers (for example: were they recruited but did not participate or was there abandonment after the initial recruitment?**

We agree and added this to Findings:

“The trial retained 11,630 (59.2%) to the primary endpoint of gender beliefs.”

2. **With reference to the same lemma, and to ensure greater accessibility in reading for a possible international reader, it would be of great help to use the official rule for punctuation in English, which provides for a comma to separate thousands and a period to separate decimal numbers. Just for example, the following cases are cited that could generate confusion:**
   a. "Abstract", paragraph "Methods", PAGE 2, LINE 2 "(n = 19,643)";
   b. "Abstract", paragraph "Methods", PAGE 2, LINE 9 "(accessed by 4·5% of the sample)": for some people who refer to international numbering rules, it could be difficult to understand that it refers to a decimal number, which could be indicated by the notation "4.5%",
   c. same problem in "Abstract", "Findings", PAGE 2 LINE 2;
      "PROCEDURES", PAGE 6, LINE 15: "Users participating in the trial were remunerated with ZAR 30 (approximately £1·50)": for some people who refer to international numbering rules, it could be difficult to understand that it refers to a decimal number, which could be indicated by the notation "£1.50%"
   d. "RESULTS", LINE 1, "We recruited 19,643 women in 31 days. As expected, 20·0%": the same correction as the previous points is proposed for similar numbering

Apologies for the formatting, which was a carry-over from the previous submission. We have now corrected throughout.

3. **"Abstract", paragraph "Methods" PAGE 2, LINE 9 "Primary outcomes were adapted (to?) validated scales of gender beliefs and past-month IPV. Secondary outcomes were identification of unhealthy relationship behaviors and depressive symptoms" would seem to be in contrast with some subsequent sentences in the rest of the document, such as: "We conducted a trial testing whether two versions of a behaviorally-informed chatbot could improve gender attitudes and skills in order to reduce IPV exposure among young women in South Africa." (INTRODUCTION, PAGE 3, LINE 37) or "Digital solutions represent an important approach to scale efforts to reduce IPV among young women. A behaviorally-informed chatbot helped women identify unhealthy relationship behaviors, improve gender equitable norms, and reduce exposure to IPV in the short-term." ("DISCUSSION", "Conclusion", PAGE** **11, LINE 1) which instead would seem more consistent with the rest of the document. If the first hypothesis indicated in "Methods" is correct, perhaps it could be written in a way that leaves no doubt for the reader;**

We thank the reviewer for this observation and have tightened the language for consistency:

Abstract: ). Primary outcomes were short-form adaptations of scales for gender attitudes (*Gender Relations Scale*) and past-month IPV (*WHO Multi-country Study Instrument)*. Secondary outcomes were identification of unhealthy relationship behaviours (*Intimate Partner Violence Attitudes Scale*) and depressive symptoms (*Patient Health Questionnaire)*.”

Introduction: “We conducted a trial testing whether two versions of a behaviorally-informed chatbot could improve gender attitudes in order to reduce IPV exposure among young women in South Africa.”

Conclusion: “A behaviorally-informed chatbot improved gender equitable attitudes and reduce exposure to IPV in the short-term.”

4. **"Abstract", "Findings" paragraph, PAGE 2, LINE 1: a reader may be interested in, at this point, knowing which tool was used to evaluate "gender beliefs";**

As noted in response to item 3, we have now included the titles of each scale.

5. **"Abstract", "Findings'' paragraph, PAGE 2, LINE 6: "Neither T1 nor T2 had a measurable effect on mental health" refers to a measurement of the absence of depression (PHQ-2). The extension of the concept of "absence of depression/anhedonia" generalized to the concept of "mental health," may lead the reader to believe that specific tools were used to evaluate "mental health," while instead, a brief screening tool was used to evaluate depression symptoms;**

Well noted, we have revised to be more consistent with the limitations of a brief, self-reported screening tool:

“Neither T1 nor T2 had a measurable effect on depressive symptoms as measured by the brief screener.”

6. **"INTRODUCTION", PAGE 3: the review section is treated very interestingly, a reader may be interested, in addition to the rightly cited information, if interventions on perpetrators/abusers have also been carried out in the same territories;**

Thank you for this suggestion and we have added to the evidence around men’s IPV perpetration:

“Similarly, few rigorously tested approaches have been shown to reduce men’s perpetration of IPV in African trials. Group-based gender training with younger men may decrease reported IPV perpetration (1), particularly if combined with economic livelihoods (2). However, other trials have had null findings for men’s IPV perpetration (3-5).”

7. **"INTRODUCTION", PAGE 3 LINE 5: "reported physical or sexual IPV" WHO defines IPV as "behaviour within an intimate relationship that causes physical, sexual or psychological harm, including acts of physical aggression, sexual coercion, psychological abuse and controlling behaviours." As can be seen from this definition, IPV already includes physical partner violence. It is understood that the citation in the text (4) reports exactly the wording of the results in table 2 (4), but probably a more effective wording can be chosen (also among those chosen in the research used as a source), to avoid that the term IPV is interpreted as exclusive for sexual violence and separate from physical violence, if both are practiced by the partner;**

This is a good clarification to make and we clarified in the Introduction:

“…in one study 53% of young women aged 14-19 years reported physical and/or sexual IPV (6).”

8. **"MATERIALS & METHODS", SUB "Treatment Arms" SUB "Pure Control arm", PAGE 4 LINE 2 "The quiz pertaining to power equity was delivered directly after onboarding and eligibility questions.": from this sentence it seems that a questionnaire on "power equity" was administered. It would be interesting to read the questionnaire: is it a previously validated questionnaire or was it constructed ad hoc? What variables does it measure? Is it one of the tools mentioned in the "Measures" paragraph?**

Thank you for this, we have altered to align with the primary outcome language of “gender attitudes”. To note, all the variables are in the section directly following this, which we hope will provide sufficient information to readers:

***Pure Control arm*** exposed eligible participants only to specific evaluation quizzes. The quiz pertaining to gender attitudes was delivered directly after eligibility screening. Follow-up quizzes three months later evaluated IPV exposure, skills of identifying harmful relationships, and the brief depressive symptoms screener.

9. **"MEASURES", PAGE 5, SUB "Gender equitable beliefs" LINE 3: What is the sample size in the validation assessment?**

We added these details:

“The aGRS is comprised of 6 items scored on Likert-type responses and had an acceptable internal consistency in the pre-trial validation with *n=*1,088 participants (Cronbach’s ⍺= 0.75).”

10. **"MEASURES", PAGE 5, SUB "Mental Health" LINE 1: "Mental health was assessed using the brief screener of depressive symptoms, the two-item Patient Health Questionnaire (PHQ-2): as already noted in point 5, a reader may have incomplete information if it is not specified that the indices used do not measure "mental health" but the absence of depression/anhedonia;**

We revised as suggested:

***“Depressive symptoms*** were assessed using a brief self-reported symptom screen: the two-item *Patient Health Questionnaire* (PHQ-2). A score of 3 or greater is suggestive of probable depression.”

11. **Also in SUB "Socio-demographic," PAGE 5 LINE 5: "The state of users' mental health was assessed through one item asking if, in the past month, they had been mostly "energised and hopeful," "just getting along," or "down and hopeless." " can lead the reader to believe that tools for the evaluation of "mental health" were used;**

Agreed, and we revised as mental distress:

“The state of participant mental distress was assessed through one item asking if, in the past month, they had been mostly “energised and hopeful”, “just getting along”, or “down and hopeless”.”

12. **"PROCEDURES", PAGE 6, LINE 6: "The trial was set up on RapidPro": an inexperienced reader may be interested in the reference to the software used and/or the organization that developed it;**

We agree and have added more detail and a relevant citation:

The trial was set up on RapidPro (open source jointly owned by UNICEF and Nyaruka) an open-sourced user management system that records user states and manages inbound and outbound messages between users and the chatbot (7).

13. **“PROCEDURES”, PAGE 6, LINE 12: “To support 18,000 users on the chatbot…”: It could be indicated that the number is approximate or the precise number of people supported by the chatbot could be provided;**

Good point, we revised without the number, since recruitment and retention are described fully elsewhere:

“To support chatbot users, careful monitoring of the database was required as it grew throughout the trial (possibly due to the design of the program, which involved a high ratio of outbound to inbound messages).”

14. **“RESULTS”, PAGE 8, LINE 6: “participants reported to have symptoms consistent with probable depression or anxiety…”: No instruments were indicated for evaluating anxiety;**

The reviewer is correct and we have revised accordingly:

“Overall, 31.7% of participants reported to have symptoms consistent with probable depression, with similar rates across arms.”

15. **“DISCUSSION”, PAGE 10, LINE 4 “The chatbot significantly improved skills around identifying unhealthy relationships, but did not alter mental health compared to the control.”**

We corrected this:

“The chatbot significantly improved skills around identifying unhealthy relationships, but did not alter depressive symptoms compared to the control.”

16. **“DISCUSSION”, PAGE 10, LINE 35 “Prevalence of mental health symptoms among this relatively large sample of young women was high…”: As observed in points 5 and 10, the concept of "mental health" is broader than the absence of elements of depression/anhedonia, which makes it complex to refer to this construct without a reader wondering how it was measured;**

Agreed and this has now been revised:

“Prevalence of depressive symptoms among this relatively large sample of young women was high, with 32% reporting symptoms consistent with probable depression on a brief screener. This rate is similar to studies nationally, which find up to 41% of young women exhibit elevated depressive symptoms (8). Neither experimental chatbot was effective at reducing depressive symptoms, nor were they associated with increased distress.”

17. **“DISCUSSION”, “Limitations”: It is suggested to further reflect on the mode of data collection (online mode), which does not guarantee: that the person who initially declared themselves is actually the one who completed the courses; that the person actually completes the activities; it is also possible that the activity is promoted/maintained by the possible receipt of financial compensation;**

Thank you, we added the first of these limitations and agree. The second proposed limitation (around the financial compensation) is not a major concern because the total reimbursement amount is far less than other research in South Africa, which typically pays ZAR 300 per questionnaire. Additionally there was no differential attrition in terms of food security by study arm, suggestive that any financial reward affected each arm of the trial similarly.

“It is possible that participants completing the quizzes were different from those who filled out baseline surveys, but we chose not to ask for South African identity numbers or private details because of confidentiality risks.”

**Reviewer #2:**

1. **the Participants in the trial were 18 or older (24yr), what their education background or education level?**

This is an important question, but we did not measure education in our sociodemographics. We added it to Limitations:

“We chose not to measure education as a socio-demographic covariate, but future studies should include this.”

2. **In this study, the literacy level could play a crucial role.**

As a text-based chatbot, literacy could indeed play a crucial role. We have added this to limitations:

“Several design characteristics of digital intervention research are worth noting as potential limitations. Low literacy may have inhibited the ability of some participants to take part in the intervention or quizzes.”

3. **In this study, mental health was assessed using the brief screener of depressive symptoms in this study, there are more complicated to assess and identify depression.**

This is an important point that we have addressed in multiple places. For example:

***“Depressive symptoms*** were assessed using a brief self-reported symptom screener: the two-item *Patient Health Questionnaire* (PHQ-2). A score of 3 or greater is suggestive of probable depression.”

“Overall, 31.7% of participants reported to have symptoms consistent with probable depression, with similar rates across arms.”

“Prevalence of depressive symptoms among this relatively large sample of young women was high, with 32% reporting symptoms consistent with probable depression on a brief screener. This rate is similar to studies nationally, which find up to 41% of young women exhibit elevated depressive symptoms (8). Neither experimental chatbot was effective at reducing depressive symptoms, nor were they associated with increased distress.”

References

1.	Jewkes R, Nduna M, et al. Impact of stepping stones on incidence of HIV and HSV-2 and sexual behaviour in rural South Africa: cluster randomised controlled trial. BMJ. 2008;337:a506.

2.	Gibbs A, Washington L, et al. Stepping Stones and Creating Futures Intervention to Prevent Intimate Partner Violence Among Young People: Cluster Randomized Controlled Trial. J Adolesc Health. 2019.

3.	Abramsky T, Devries K, et al. Findings from the SASA! Study: a cluster randomized controlled trial to assess the impact of a community mobilization intervention to prevent violence against women and reduce HIV risk in Kampala, Uganda. BMC Med. 2014;12(1):122.

4.	Hossain M, Zimmerman C, et al. Working with men to prevent intimate partner violence in a conflict-affected setting: a pilot cluster randomized controlled trial in rural Cote d'Ivoire. BMC public health. 2014;14:339.

5.	Pettifor A, Lippman SA, et al. Community mobilization to modify harmful gender norms and reduce HIV risk: results from a community cluster randomized trial in South Africa. J Int AIDS Soc. 2018;21(7):e25134.

6.	Shamu S, Gevers A, et al. Prevalence and risk factors for intimate partner violence among Grade 8 learners in urban South Africa: baseline analysis from the Skhokho Supporting Success cluster randomised controlled trial. Int Health. 2016;8(1):18-26.

7.	UNICEF. Case Study: RapidPro 2019 [Available from: <https://www.unicef.org/evaluation/media/976/file/RapidPro.pdf>.

8.	Das-Munshi J, Lund C, et al. Mental health inequalities in adolescents growing up in post-apartheid South Africa: cross-sectional survey, SHaW study. PloS one. 2016;11(5):e0154478.
